# *“No-Primer”* Resin Cementation of Lithium Disilicate Ceramic: A Microtensile Bond Strength Evaluation

**DOI:** 10.3390/ma17010137

**Published:** 2023-12-27

**Authors:** Mohamed M. Awad, Feras Alhalabi, Abdulaziz Abdullah Alanazi, Abdulaziz Ali Alanazi, Ghanem Abdullah Alshahrani, Cheryl Fu, Refal Saad Albaijan, Rana Alkattan, Amr S. Fawzy

**Affiliations:** 1 UWA Dental School, University of Western Australia, 17 Monash Avenue, Nedlands, WA 6009, Australia; 21295218@student.uwa.edu.au (C.F.) amr.fawzy@uwa.edu.au (A.S.F.); 2 Department of Conservative Dental Sciences, College of Dentistry, Prince Sattam Bin Abdulaziz University, Al-Kharj 11942, Saudi Arabia; 3 College of Dentistry, Prince Sattam Bin Abdulaziz University, Al-Kharj 11942, Saudi Arabia; 437050633@std.psau.edu.sa (A.A.A.); 437050940@std.psau.edu.sa (A.A.A.); ghanem.shh@gmail.com (G.A.A.); 4 Department of Prosthetic Dental Sciences, College of Dentistry, Prince Sattam Bin Abdulaziz University, Al-Kharj 11942, Saudi Arabia; r.albaijan@psau.edu.sa; 5 Restorative and Prosthetic Dental Sciences, College of Dentistry, King Saud Bin Abdulaziz University for Health Sciences, Riyadh 11545, Saudi Arabia; kattanr@ksau-hs.edu.sa; 6 King Abdullah International Medical Research Center, Ministry of National Guard Health Affairs, Riyadh 11545, Saudi Arabia

**Keywords:** resin-ceramic adhesion, microtensile bond strength, self-adhesive cement, long-carbon-chain silane

## Abstract

Objective: The objective of this study was to evaluate the resin-ceramic adhesion of a long-carbon-chain silane (LCSI)-containing resin cement. Methods: Polished lithium disilicate ceramic discs were etched with hydrofluoric acid and randomly assigned into four groups; (PSAP), cemented using a silane-free resin cement with no prior priming; (PSAP-S), primed using a silane-containing primer before cementation using a silane-free resin cement; (PSAU), cemented using a LCSI-containing resin cement with no prior priming; (PSAU-S), primed as for the group (PSAP-S) and cemented using a LCSI-containing resin cement. The cemented blocks were sectioned into microbeams. The resin-ceramic microtensile bond strength (μTBS) was measured at 1 week and after thermocycling. The failure modes of the tested microbeams were evaluated. Results: The μTBS of the LCSI-containing and silane-free resin cements, either with or without a prior priming step, did not significantly differ. The adhesion of the LCSI-containing resin cement to lithium disilicate ceramic, either with or without a prior priming step, did not significantly deteriorate after artificial aging. Conclusions: The long-carbon-chain silane (LCSI) monomer incorporated in the resin cement eliminated the need for a silane priming step of a hydrofluoric acid-etched lithium disilicate ceramic.

## 1. Introduction

The longevity of ceramic restorations can be influenced by the cementation protocol utilized to achieve durable adhesion between the ceramic restoration and the underlying structure. Although different categories of dental cements can be used for such a purpose, resin cements are more suitable for the cementation of glass-ceramic restorations due to their mechanical, on-demand curing, and esthetic properties [1]. Resin cements are generally composed of a resin matrix (monomers) with modified rheological properties, fillers, pigments, opacifiers, and a polymerization system. Resin-ceramic cementation is influenced by the composition of the resin cement and the microstructure of the ceramic material [2].

Glass-ceramic materials are most used to fabricate indirect esthetic restorations [3]. Of the commercially available glass-ceramic materials, lithium disilicate ceramic (LDC) has been identified as the material of choice for modern prosthetic dentistry [4]. It can be supplied in fully crystallized, partly crystallized, or heat-pressed forms [5]. The microstructure/composition of LDC comprises a glass-based matrix (glassy phase) reinforced by crystals such as lithium disilicate [5,6]. Hence, a hydrofluoric acid (HF) etchant can be used to etch the ceramic glassy phase to create prominent topographic changes in the ceramic surface for better adhesion [7]. Subsequently, a post-etching priming step using an organofunctional trialkoxysilane (silane)-containing primer is recommended [8]. Silane is a bi-functional monomer that bonds chemically to methacrylate-based resin cement via its organofunctional (methacrylate) group while its silanol group bonds to the ceramic surface. The organofunctional and hydrolysable groups of the silane monomer are linked by a hydrocarbon chain that is critical for the chemical stability and hydrophobicity of the silane monomer. Silane-containing primers indicated for dental use contains less than 5% of silane monomers, such as γ-methacryloxypropyltrimethoxysilane (γ-MPTS), diluted and dissolved in large amounts of solvents like ethanol, methyl ethyl ketone, and acetone in addition to water [8]. Upon the clinical application of such primers onto the ceramic surface, a condensation reaction between silane and the ceramic surface results in water formation [9]. Thus, air-drying the primed ceramic surface is pivotal to eliminate the excess solvent and water, otherwise a loose film of silane film will be formed on the ceramic surface, deteriorating the obtained resin-ceramic adhesion [10]. The air-drying procedure may be insufficient to completely evaporate the required amount of solvent and water. Therefore, using warm air (45 ± 5 °C) has been a suggested approach to enhance the effectiveness of solvent and water evaporation from silane-containing primers [10].

To simplify the priming step of glass-ceramic materials, silane-containing adhesives have been introduced. However, such adhesives failed to be reliable alternatives to the commercially available silane-containing primers [11,12,13]. Recently, a novel resin cement containing trimethoxysilyl long-chain alkyl methacrylate monomer (long-carbon-chain silane (LCSI)) was introduced to simplify the cementation procedures of LDC restorations. Such a resin cement has been claimed to effectively bond (adhesion) to LDC with no need for a separate (silane priming) silanization step, saving chair-side time and decreasing the chances for clinical errors. LCSI-containing resin cement showed promising results, improving the resin-ceramic macro-shear bond strength, with or without silane priming [14]. However, the macro-shear bond strength test is associated with uneven stress distribution at the interface and more cohesive failures within the substrate, which minimizes the discriminative ability of such a method. Studies utilizing micro-bond strength testing methods, such as the microtensile bond strength (µTBS) test, can provide a more precise evaluation of the “*no-primer*” cementation protocol of LDC. Therefore, the effect of a LCSI-containing resin cement, with or without a prior silane priming step, on the resin-ceramic µTBS was evaluated. It was hypothesized that (1) there was no difference between the resin-ceramic µTBS of LCSI-containing and silane-free resin cements; and (2) the silane-containing universal primer would not significantly influence the resin-ceramic µTBS of either the LCSI-containing or silane-free resin cement.

## 2. Materials and Methods

Table 1 describes the ceramic material, resin cement, and silane-containing primer utilized in this study as well as their compositions.

### 2.1. Specimens’ Preparation

CAD/CAM blocks (GC Initial LiSi Block, GC Corporation, Tokyo, Japan) provided in a fully crystallized form were employed in the current study. The CAD/CAM blocks were horizontally cut under water coolant into ~5 mm-thick discs using a 6-inch diamond cutting blade installed on an automatic precision cutting saw operated at a high speed. For each group, the top and bottom surfaces of the obtained ceramic discs were polished under water using 600-grit silicon carbide papers attached to a grinder/polisher machine (MetaServ^®^ 250 grinder-polisher machine, Buehler, Lake Bluff, IL, USA) operated at 150 revolutions per minute (rpm). This was followed by ultrasonic cleaning the polished discs in distilled water.

**Figure 1:**
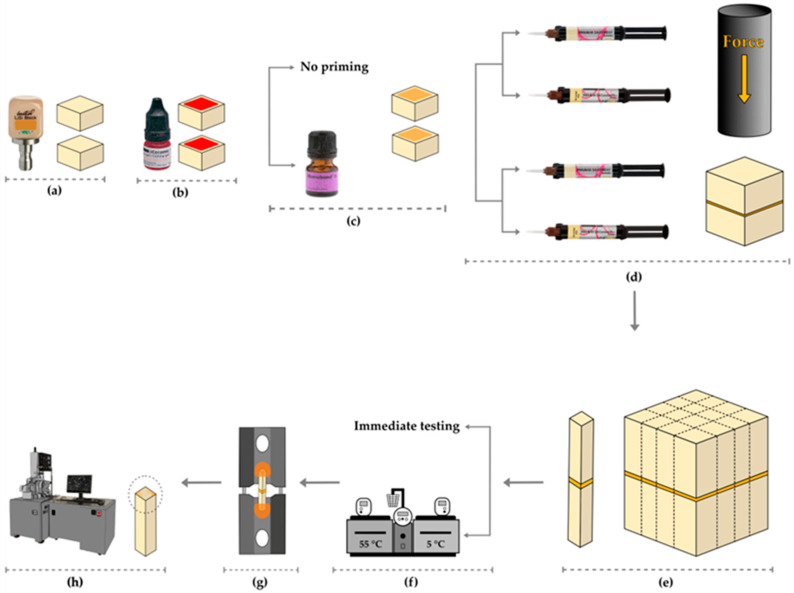
Schematic diagram describing the study design, specimen preparation, and testing. (**a**) The sectioning of the lithium disilicate (LDC) CAD/CAM blocks; (**b**) etching of the LDC discs using hydrofluoric acid; (**c**) LDC priming step; (**d**) cementing of two ceramic discs with the same surface treatment using either silane-free resin cement or LCSI-containing resin cement; (**e**) sectioning of the cemented ceramic blocks; (**f**) artificial aging by thermocycling; (**g**) μTBS test; (**h**) failure mode assessment.

The ceramic surface was surface treated with a ~5% hydrofluoric acid ceramic etchant (IPS Ceramic Etching Gel, Ivoclar Vivadent, Schaan, Liechtenstein) for 20 s as recommended by the manufacturer of the ceramic material, subsequently neutralized with sodium carbonate and calcium carbonate powder, washed thoroughly with water, cleaned ultrasonically with distilled water, and subsequently air-dried. The HF-etched ceramic discs were randomly assigned to four experimental groups (*n* = 6 pairs of discs/group) based on the priming step performed and the resin cement utilized (Figure 1). In the first group (PSAP), the ceramic discs were not primed and then cemented using a silane-free resin cement (Panavia SA Cement Plus). In the second group (PSAP-S), a silane-containing universal primer (Monobond N) was used to prime the top surface of the ceramic disc. According to the manufacturer’s instructions, the primer solution was applied and maintained for 60 s onto the ceramic surface, then it was effectively air-dried, and the ceramic discs were cemented using Panavia SA Cement Plus, Kuraray Noritake. In the third group (PSAU), the ceramic discs were not primed and then cemented using a LCSI-containing resin cement (Panavia SA Cement Universal). In the fourth group (PSAU-S), the ceramic discs were primed as described for group 2 and then cemented using Panavia SA Cement Universal, Kuraray Noritake. For each group, two discs were vertically aligned and then cemented under a 1 kg force for 1 min using a custom-made cementation jig (Figure 1). The excess resin cement was carefully eliminated. The cemented block interface was light-cured for 40 s from four directions using a light-curing device (Elipar^TM^ S10, 3M ESPE, St. Paul, MN, USA) worked at ~900 mW/cm^2^, as verified by a digital radiometer. The light-curing tip was positioned at 1 ± 0.2 mm to the target area. The cemented blocks were then stored for 48 h in a distilled water bath at 37 °C.

### 2.2. μTBS Evaluation

The specimens were sectioned across the interface into (~1 mm × ~1 mm) microbeams using a 4-inch diamond cutting blade installed on a precision saw (IsoMet 1000 Precision Cutter, Lake Bluff, IL, USA) operated at a low speed (Figure 1). The pretest failures that occurred during the micro-sectioning were noted. Half of the microbeams obtained from each specimen were stored for 1 week in a distilled water bath at 37 °C in an incubator (HeraTherm Oven, TheromScientific, Waltham, MA, USA) and then used for the evaluation of the resin-ceramic *μTBS.* The other half underwent artificial aging via 10k thermocycling. In each cycle, the microbeams were immersed into a 5 °C followed by a 55 °C distilled water bath. The dwell time was 30 s, and the transfer time was 5 s. A digital micrometer (Mitutoyo, Kanagawa, Japan) was utilized to calculate the cross-sectional surface area of the microbeams prior to testing. Each microbeam was glued onto a custom-made stainless steel μTBS jig using a cyanoacrylate glue, ensuring that the microbeam was aligned parallel to the long axis of the μTBS jig. The μTBS jig was then installed on a universal testing machine (Instron 5965, Instron Corporation, Norwood, MN, USA) equipped with a 1000 N static load cell. A tensile force was applied to the microbeam at a constant 0.5 mm per min crosshead speed until failure. The μTBS (in megapascals (MPa)) was calculated using the Bluehill^®^2 Software, Instron Corporation, Norwood, MN, USA, considering the maximum force (N) recorded at failure and the premeasured microbeam’s cross-sectional surface area (mm^2^).

### 2.3. Failure Mode Assessment

The tested microbeams were collected and viewed using a stereo microscope (Hirox Co., Ltd., Tokyo, Japan) at a 15× magnification to assess the failure mode patterns. The microbeams with failure modes that could not be identified using the stereo microscope were cleaned ultrasonically in distilled water and then immersed in ascending concentrations of ethanol to dehydrate. The dehydrated microbeams were vertically mounted and gold sputtered before viewing the microbeam’s interfacial area at a higher magnification (120×) using a field emission scanning electron microscope (Verios, FEI, Hillsboro, OR, USA) worked at 10 kV. The observed failure mode patterns were categorized as a cohesive failure within the substrate, interfacial or adhesive failures at the interface, or mixed failures involving a partial adhesive failure in addition to either a failure/fracture within the substrate or a cohesive failure within the interface.

### 2.4. Statistical Analysis

The specimen (cemented block) dependency was considered in the statistical analysis of the μTBS data. Accordingly, the μTBS values recorded for the microbeams obtained from the same specimen were averaged. The pretest failures (PTFs) were included as 0 MPa in the statistical analysis. The normality of the μTBS data was determined using the Shapiro-Wilk test. A two-way analysis of variance (ANOVA) test (α = 0.05) was utilized in the statistical analysis of the µTBS data to evaluate the effect of the priming step, the type of resin cement, as well as their interaction on the resin-ceramic µTBS. Pair-wise group comparisons were done using Tukey’s multiple comparisons test. Within each group, a dependent *t*-test was utilized to compare the resin-ceramic µTBS recorded either at 1 week or after 10k thermocycling (aged). A 5% significance level was considered in all the statistical analyses. R 4.1.3, R project for Statistical Computing, Vienna, Austria was used to perform all the all the statistical analyses.

## 3. Results

### 3.1. μTBS

**Figure 2:**
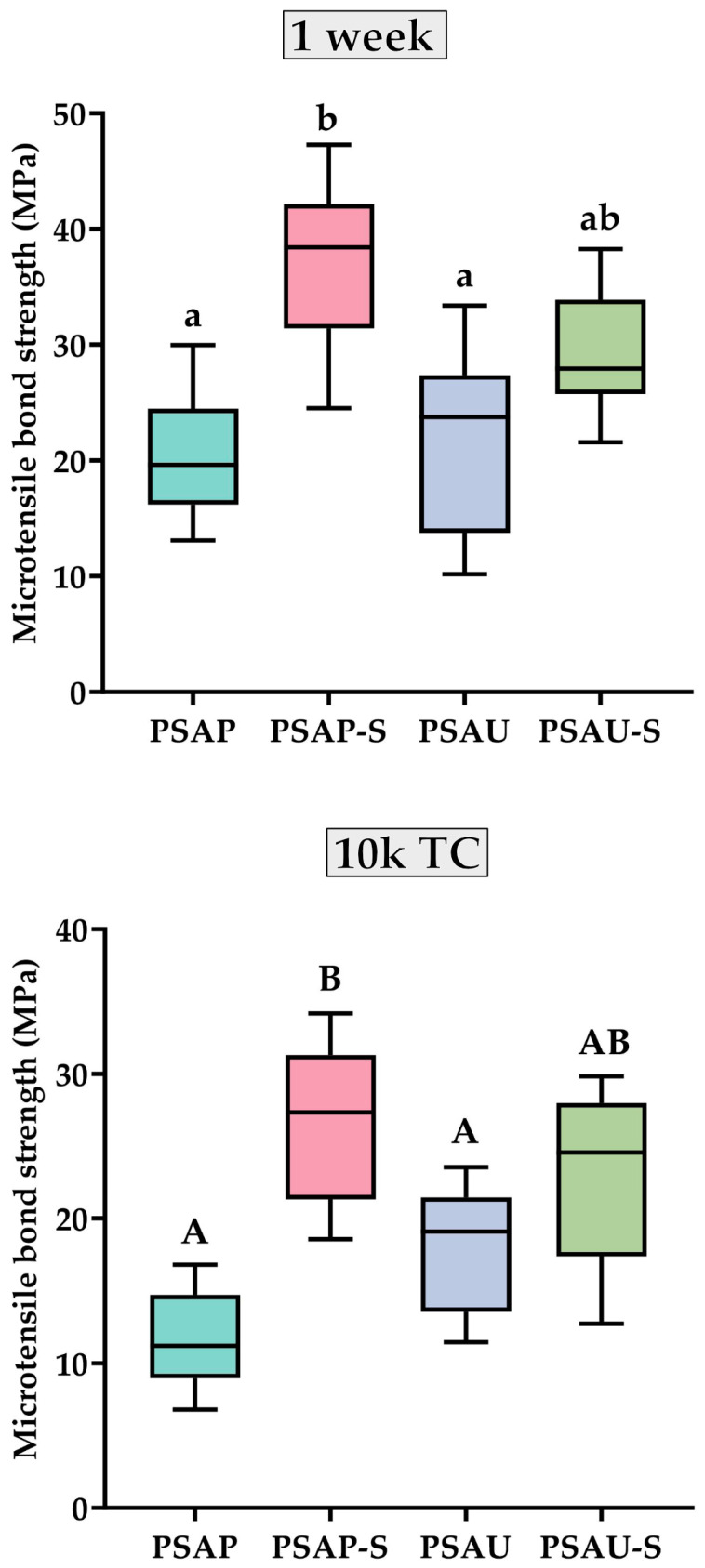
Box-whisker plots illustrating the minimum, maximum, interquartile range, and median resin-ceramic microtensile bond strength (µTBS) expressed in megapascals (MPa) for all the experimental groups, measured either at 1 week or after artificial aging by thermocycling (10k TC). Statistically significant differences between the experimental groups recorded either at 1 week or thermocycling are indicated with different lower-case and upper-case superscript letters, respectively.

The mean μTBS, standard deviation, number of tested microbeams, and PTFs for each group are presented in Table 2. Furthermore, the minimum, maximum, interquartile range, and median μTBS for each group are graphically presented in Figure 2.

At 1 week and after thermocycling, the difference between the silane-free resin cement (PSAP) and LCSI-containing resin cement (PSAU) was statistically insignificant either with or without a prior priming step using a silane-containing primer. The results of the two-way ANOVA of the µTBS data at 1 week and after thermocycling are presented in Table 3 and Table 4, respectively. The resin cement factor did not significantly influence the resin-ceramic µTBS either at 1 week or after 10k TC. In contrast, the priming factor significantly affected the resin-ceramic µTBS. Priming the HF-etched lithium disilicate ceramic material using a silane-containing universal primer (MBN) significantly improved the bonding of PSAP to lithium disilicate material. However, it did not have a significant effect on the μTBS of the LCSI-containing resin cement (PSAU) recorded either at 1 week or after thermocycling. Interestingly, the adhesion of PSAU to the lithium disilicate ceramic material, either with or without a prior priming step, did not significantly deteriorate after thermocycling. In contrast, thermocycling significantly reduced the bonding (adhesion) strength of the PSAP and PSAP-S groups.

### 3.2. Failure Mode Evaluation

**Figure 3:**
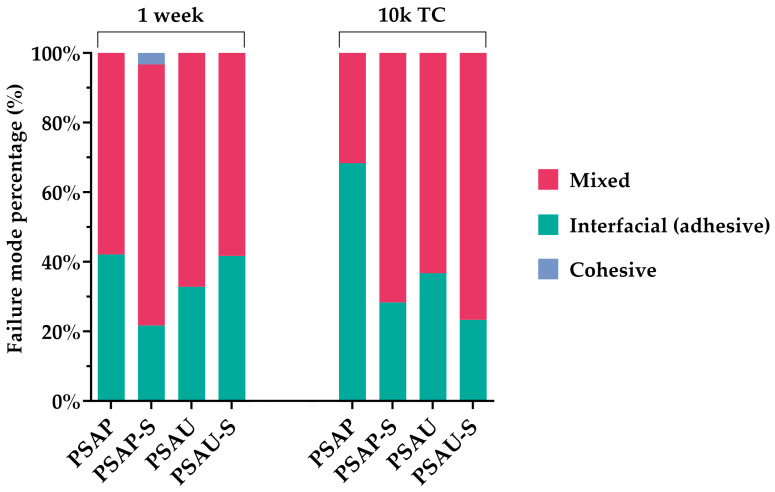
The percentage of the different failure modes of the tested microtensile bond strength (μTBS) microbeams of each experimental group reported either at 1 week or after thermocycling.

**Figure 4:**
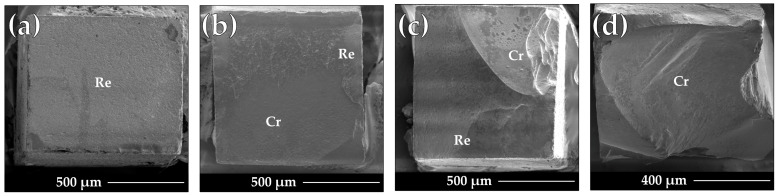
Scanning electron microscope (SEM) images of the tested microbeams, illustrating the different failure mode patterns recorded. (**a**) Adhesive failure; (**b**,**c**) mixed failures; (**d**) cohesive failure in the ceramic; Cr: ceramic; Re: resin cement.

Figure 3 illustrates the frequencies (percentages) of the different failure modes noticed for the microbeams tested for μTBS either at 1 week or after thermocycling (10k TC). Except for the PSAP group after thermocycling, mixed failures showed the highest incidence in all the other groups either at 1 week or after thermocycling. Interfacial (adhesive) failures were the second most frequent in all the groups, except for the PSAP group after thermocycling. Cohesive failures were the least predominant failures and were only detected in the PSAP-S group at 1 week. Pretest failures (PTF) were recorded during sectioning only for the groups PSAP and PSAU at 3 and 2 microbeams, respectively. SEM images representing the different failure mode patterns observed are illustrated in Figure 4.

## 4. Discussion

The main aim of the current study was to determine whether the LCSI-containing resin cement could effectively bond with a HF-etched ceramic surface without a prior silane priming step. Except for the silane (LCSI) content, the monomers content of the two resin cements (PSAP and PSAU) used in this study are identical in composition, according to their manufacturer. This minimized the possible effects of the other resin cement components such as the resin matrix, fillers, or initiator/accelerator system on the obtained resin-ceramic adhesion. A μTBS test was utilized in this study due to its precision and discriminative ability compared to macro bond strength tests [15]. The laboratory guidelines provided by Armstrong et al., 2017 for the evaluation of μTBS were applied in this study [16]. Although those guidelines were set primarily for the evaluation of resin-dentin and resin-enamel μTBS, they could be also applied for evaluating the bond (adhesion) strength of dental ceramics. Additionally, an intra-oral cementation procedure was simulated by cementing the ceramic discs under force rather than applying the resin cement onto the ceramic surface passively [13,17]. This was thought to ensure intimate contact between the resin cement paste and the ceramic surface and to minimize chances for void formation within the resin cement. Artificial aging via 10k thermocycling of the microbeams was performed to simulate one year of clinical service [18].

The μTBS results indicated that the two resin cements tested did not show statistically significant differences, either with or without a separate salinization step, at 1 week and after thermocycling. However, the silanization step significantly influenced the μTBS of the silane-free resin cement at 1 week and after thermocycling. Accordingly, the first null hypothesis had to be accepted, and the second null hypothesis had to be rejected. In contrast to silane-containing adhesives, which could not effectively prime the glass-ceramic materials [11,12] due to the chemical instability of silane monomers within the acidic adhesive solution [19], the LCSI-containing resin cement tested presented durable μTBS to the LDC with or without silane priming. In fact, adding silane to the hydrophobic, not the hydrophilic (acidic), paste of PSAU maintained the priming efficiency of the silane monomers [14]. Although the hydrolysis of the silane monomer could be challenged by a lack of water in the resin cement formulation, it was speculated that the water absorbed on the HF-etched ceramic might have influenced silane hydrolysis (activation) [20]. In addition, a 29Si nuclear magnetic resonance (NMR) analysis of the silane-containing resin cement indirectly confirmed silane hydrolysis and the formation of silanol groups upon the mixture of the two resin cement pastes (paste A and paste B) [14]. A condensation reaction between the silanol groups of the hydrolyzed silane and the hydroxyl groups formed onto the ceramic surface after HF etching was expected to promote the chemical bonding of the LCSI-containing resin cement to the LDC [7,21]. Thus, in this study, the resin-ceramic μTBS of PSAU was not affected by the prior silane priming step. On the other hand, the bonding of the silane-free resin cement (PSAP) was significantly improved with a separate silane priming step. This can be explained by the priming efficacy of the universal (silane-containing) primer applied prior to the resin cementation. As far as such a primer was applied correctly and air-dried sufficiently, the formation a consistent silane film on the primed ceramic surface could promote chemical bonding between the ceramic surface and methacrylate-based resin cement [12,13,21,22]. Apparently, resin-ceramic bonding can be attributed to silane priming either before (γ-MPTS primer) or simultaneously during the application of LCSI-containing resin cement. In fact, LCSI primers have similar glass-ceramic priming efficacies as γ-MPTS primers. However, LCSI monomers are more chemically stable because the long hydrocarbon chains can reduce the possibility of silane self-condensation [23]. In addition, LCSI enhanced the mechanical properties of an experimental composite when LCSI was utilized to promote the bonding of fillers to the resin matrix [24]. The other components of the two resin cements tested, such as the 10-methacryloyloxydecyl dihydrogenphosphate (10-MDP) functional monomer, were believed to play an insignificant role in bonding to the glass-based ceramics [25]. Although the percentage of the LCSI monomer was not revealed by the manufacturer, the similarity in the composition of both resin cements, particularly the initiator system and monomers incorporated, might have elucidated the similar performance of the two resin cements tested. PSAU presented no significant reduction in the adhesion after thermocycling, either with or without a silane priming step, which might be explained by the optimized priming capacity and chemical adhesion of such a resin cement to LDC. Except for PSAP (without silane priming) after thermocycling, all the other groups exhibited a clinically acceptable mean bond strength. However, this should be considered carefully as the real value of adhesion testing relies on the comparison between the groups more than the actual mean bond strength recorded as they can be greatly affected by a multitude of factors, including the sample preparation, the experimental setup, and the used equipment.

Despite the precision and discriminative nature of the μTBS test employed in this study, the interpretation and analysis of the μTBS data should be attempted considering the corresponding failure modes. For example, the PTFs should be recorded and considered in a statistical analysis. The incidence of the PTFs can be an indicator for suboptimal adhesion. Meanwhile, the outcome of the statistical analysis for the μTBS data might be influenced if the values of the cohesively failed microbeams are censored or considered in the statistical analysis [26]. The structure of the microbeams prepared and tested for μTBS probably affected the failure modes observed in this study. To specify, each microbeam consisted of two ceramic parts cemented together. This might have reduced the incidence of cohesive failures in the substrate (ceramic) due to its high mechanical properties [13]. The fractographic assessment of the tested microbeams indicated that, except for the PSAP group after aging, which presented the highest incidence of adhesive failures, all the microbeams of the other groups showed mixed failures.

Overall, the outcome of this study demonstrated that LCSI-containing resin cement (PSAU) showed a promising adhesive performance to LDC. LCSI-containing resin cement is expected to bond effectively to other glass-ceramic materials, such as leucite reinforced or feldspathic ceramic materials, after HF acid etching. While the outcome of the current study is in accordance with previous research [14], the findings of the current study should be considered with caution due to the study’s limitations, which involve the lack of an in-depth characterization of the reaction between LCSI-containing resin-cement (PSAU) and LDC. Furthermore, the physical and mechanical properties, in addition to the polymerization kinetics of the two resin-cements tested, should be thoroughly investigated as they are be correlated with the adhesion strength of such resin-cements [27,28].

## 5. Conclusions

Within the confines of the in-vitro setup of this study, the LCSI monomer incorporated into the resin cement eliminated the need for a silane priming step of a HF-etched LDC material. Although the resin-ceramic adhesion strength of both the LCSI-containing and silane-free resin cements tested did not significantly differ, a silane priming step was still required prior to the cementation of a HF-etched LDC material using a silane-free resin cement. Further research involving a multi-factorial simulation of the intra-oral conditions and cementation of glass-ceramic restorations to abutment teeth using a LCSI-containing resin cement is suggested.

## Figures and Tables

**Table 1:** Materials used in the study.

Material	Code	Composition
GC Initial LiSi Block, GC corporation, Tokyo, Japan	-	Silicon dioxide, phosphorus oxide, potassium oxide, aluminum oxides, titanium oxide, and cerium oxide.
Panavia SA Cement Plus, Kuraray Noritake, Tokyo, Japan	PSAP	Paste A composition: 10-methacryloyloxydecyl dihydrogen phosphate, bisphenol A glycidyl methacrylate, triethylene glycol dimethacrylate, 2-hydroxyethyl methacrylate, fillers, initiator, pigment, and other components. Paste B composition: Methacrylate monomer, fillers, accelerator, pigment, other components.
Panavia SA Cement Universal, Kuraray Noritake, Tokyo, Japan	PSAU	Paste A composition: 10-methacryloyloxydecyl dihydrogen phosphate, bisphenol A glycidyl methac-rylate, triethylene glycol dimethacrylate, 2-hydroxyethyl methacrylate, fillers, initiator, pigment, and other components. Paste B composition: Methacrylate monomer, fillers, accelerator, pigment, long-chain silane, and other components.
Monobond N, Ivoclar Vivadent, Schaan, Liechtenstein	MBN	Alcohol, silane methacrylate, phosphoric acid methacrylate, and disulfide methacrylate.

**Table 2:** Mean ± standard deviation (SD) resin-ceramic microtensile bond strength (µTBS) expressed in megapascals (MPa) for the experimental groups at 1 week and after thermocycling (10k TC).

	µTBS (1 Week)		µTBS (10k TC)	
	Mean ± SD (MPa)	^1^ PTF/^2^ *n*	Mean ± SD (MPa)	PTF/*n*
PSAP	20.35 ± 5.68	3/57	11.62 ± 3.47 *	0/60
PSAP-S	37.12 ± 7.62	0/60	26.67 ± 5.68 *	0/60
PSAU	21.89 ± 8.20	2/58	18.04 ± 4.42	0/60
PSAU-S	29.20 ± 5.63	0/60	23.01 ± 6.29	0/60

^1^: Pretest failure; ^2^: Number of tested microbeams per group. *: Indicates a statistically significant difference within each group (the same row) after artificial aging by thermocycling (10k TC).

**Table 3:** The results of the two-way ANOVA statistical analysis for the μTBS data at 1 week.

	Degrees of Freedom	Sum Squares	Mean Square	F-Value	*p*-Value
Resin cement	1	61.2	61.2	1.293	0.269
Priming	1	870.1	870.1	18.390	<0.001 *
Resin cement × Priming	1	134.2	134.2	2.836	0.108
Residuals	20	946.2	47.3		

*: Indicates a statistically significant effect.

**Table 4:** The results of the two-way ANOVA statistical analysis for the μTBS data recorded after thermocycling.

	Degrees of Freedom	Sum Squares	Mean Square	F-Value	*p*-Value
Resin cement	1	11.3	11.3	0.437	0.516
Priming	1	600.3	600.3	23.219	<0.001 *
Resin cement × Priming	1	152.6	152.6	5.904	0.0246 *
Residuals	20	517.1	25.9		

*: Indicates a statistically significant effect.

## Data Availability

The data presented in this study are available upon request from the corresponding authors.
